# Nondestructive Analysis of Debonds in a Composite Structure under Variable Temperature Conditions

**DOI:** 10.3390/s19163454

**Published:** 2019-08-07

**Authors:** Shirsendu Sikdar, Abhishek Kundu, Michał Jurek, Wiesław Ostachowicz

**Affiliations:** 1 Institute of Fluid-Flow Machinery, Polish Academy of Sciences, 14, Fiszera Street, 80-231 Gdansk, Poland; 2 Cardiff School of Engineering, Cardiff University, The Parade, Queen’s Building, Cardiff CF24 3AA, UK; 3 Department of Structural Mechanics, Ignacy Lukasiewicz Rzeszów University of Technology, 2 Poznańska Street, 35-959 Rzeszów, Poland

**Keywords:** adhesive bonds, carbon-fibre composites, debond, guided wave, infrared-thermography, piezoelectric transducer

## Abstract

This paper presents a nondestructive analysis of debonds in an adhesively-bonded carbon-fibre reinforced composite structure under variable temperature conditions. Towards this, ultrasonic guided wave propagation based experimental analysis and numerical simulations are carried out for a sample composite structure to investigate the wave propagation characteristics and detect debonds under variable operating temperature conditions. The analysis revealed that the presence of debonds in the structure significantly reduces the wave mode amplitudes, and this effect further increases with the increase in ambient temperature and debond size. Based on the debond induced differential amplitude phenomenon, an online monitoring strategy is proposed that directly uses the guided wave signals from the distributed piezoelectric sensor network to localize the hidden debonds in the structure. Debond index maps generated from the proposed monitoring strategy show the debond identification potential in the adhesively-bonded composite structure. The accuracy of the monitoring strategy is successfully verified with non-contact active infrared-thermography analysis results. The effectiveness of the proposed monitoring strategy is further investigated for the variable debond size and ambient temperature conditions. The study establishes the potential for using the proposed damage index constructed from the differential guided wave signal features as a basis for localization and characterization of debond damages in operational composite structures.

## 1. Introduction

Carbon-fibre reinforced composite (CFRC) structures are widely used in the aerospace, automotive and marine industries, due to their effective fire and corrosion resistance, acoustic insulation, high stiffness/weight ratios, construction flexibilities and lightweight advantages [1,2,3,4]. In several construction requirements, two or more such composite laminates are bonded with epoxy adhesives and debonds can occur at such adhesive interfaces, due to temperature fluctuations, improper handling, impact, fatigue and ageing [5,6,7,8,9]. Hence, fast and effective nondestructive evaluation and structural health monitoring (SHM) strategies are required to detect such hidden debonds under variable ambient temperature conditions, in order to avoid catastrophic failures while the structure is in-service [9,10]. Moreover, study of temperature effects on debond response is also important, as the change in temperature can significantly affect the results from such monitoring strategies [11].

Ultrasonic guided wave propagation-based SHM strategies have proven their potential to effectively identify hidden defects in complex layered materials [7,8,9,10,11,12,13,14,15]. As the piezoelectric transducer (PZT)-induced guided wave propagation-based SHM techniques offer long-distance inspection potential due to the penetration capacity into different layers in the composites with less attenuation [12,13,14,15]. In guided wave propagation-based techniques, analysis of dispersion characteristics is crucial for the development of an effective SHM strategy using PZT actuator-sensor networks [12].

In several studies [8,15,16,17], the finite element method (FEM) based numerical simulations of guided wave propagation in composite structures is presented. The FEM simulation has proven its potential to effectively model the ultrasonic guided wave propagation phenomenon in composite/metallic structures.

A global matrix formation-based theoretical model is presented for high-frequency wave propagation in composite structures [18]. The two-dimensional (2D) model has proven its potential to effectively represent the modal characteristics of the propagating guided signal in multi-layered composite structures.

Active thermography measurement-based non-contact NDE techniques for layered structures are also proposed by many authors [19,20,21,22,23,24,25,26,27,28]. The infrared thermography (IRT) test based NDE techniques is applicable to a wide range of materials, including CFRCs [22,23,24]. Moreover, the IRT test is applicable in production as well as in maintenance works, which makes it a versatile and flexible technique compared to other conventional NDE technologies. In active IRT, the infrared (IR) camera receives different levels of IR radiation from the surface of the target structure, which has been thermally stimulated and based on the distribution of the recorded radiation an image called ‘*thermogram*’ is generated. The existence of structural defect creates different thermal conduction in the material that affects the heat flow. In the process, the existence of discontinuities (e.g., cracks, debondings, indentations) in the target structure will make it colder or warmer at a different ratio that will ultimately lead to the appearance of different thermal contrasts in the simulated thermograms [24,25,26,27,28].

The present study is devoted towards the development of a reliable, fast and efficient online SHM strategy to identify the hidden debonds in an adhesively-bonded carbon-fibre composite structure (ACCS) under variable operating temperature conditions. In the process, combined experimental investigations in the laboratory and FEM-based 3D numerical simulations in ABAQUS are carried out for the analysis of ultrasonic guided wave propagation in a sample ACCS under different temperature conditions are carried out using an edge-reflection free sparse network of PZTs. A probabilistic analysis based SHM strategy is also prepared in MATLAB, in order to detect debonds under variable ambient temperature conditions. The proposed monitoring strategy uses the differential changes in guided wave signals obtained from the pre-assigned sensor network. The effectiveness of the SHM strategy is cross-verified with the non-contact active IRT analysis results.

## 2. Experimental Analysis

Laboratory experiments are conducted on a sample ACCS (25 cm × 25 cm × 0.7 cm) with a purposely-built circular debond region at the bond-interphase. The debond diameter (φ) is measured to be 0.8 cm (approximately). The sample ACCS is composed of two 0.344 cm thick eight-layer twill (±45^°^) CFRC laminates, and the laminates are bonded with a 0.012 cm (approximate) thin layer of epoxy adhesive. The experimental studies on PZT network-based guided wave propagation and the non-contact IRT based inspections are separately carried out on a sample ACCS as described below.

### 2.1. Experimental Analysis Using Guided Wave Propagation

**Figure 1:**
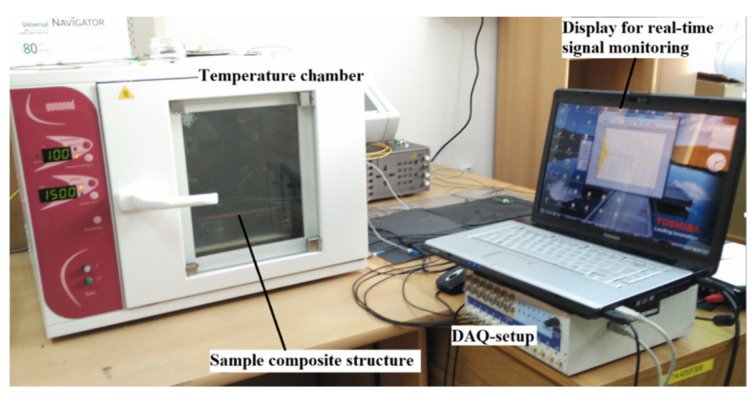
Experimental setup for the temperature-dependent analysis of debond effects on guided waves in the adhesively-bonded carbon-fibre composite structure (ACCS).

In order to study the characteristics of ultrasonic guided wave propagation and its interaction with debond in ACCS under variable temperature conditions, a series of laboratory experiments has been carried out using a temperature-control chamber and a pre-defined actuator–sensor network of PZTs assigned on the ACCS surface. A multi-channel signal generator-cum-data acquisition (DAQ) instrument is deployed to operate (guided wave actuation and reception) the assigned PZTs. A detailed description of the experimental setup is shown in Figure 1. 

**Figure 2:**
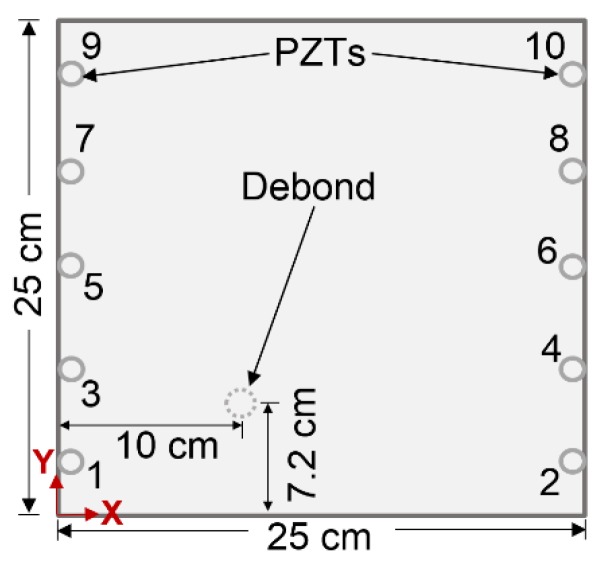
Schematic representation of the assigned piezoelectric transducer (PZT) network on the sample ACCS.

The PZT (actuator/sensor) arrangement on the sample surface against the debond is described in Figure 2 and the coordinates (x,y) of the PZTs are specifically given in Table 1. A selected set of operating temperatures (0 °C, 20 °C, 60 °C, and 100 °C) is selected for the temperature-chamber, in order to carry out the temperature dependent experiments.

**Figure 3:**
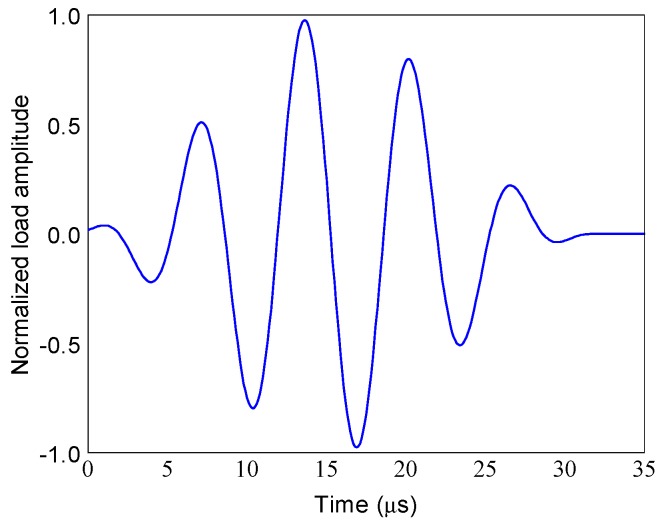
Actuator signal: 150 kHz five-cycle tone-burst sine pulse.

In the guided wave propagation-based analysis of ACCS, a Hanning-window modulated 150 kHz five-cycle tone-burst sine pulse, as shown in Figure 3, is applied as actuation signals for the PZTs.

### 2.2. Experimental Analysis Using Active IRT Test

**Figure 4:**
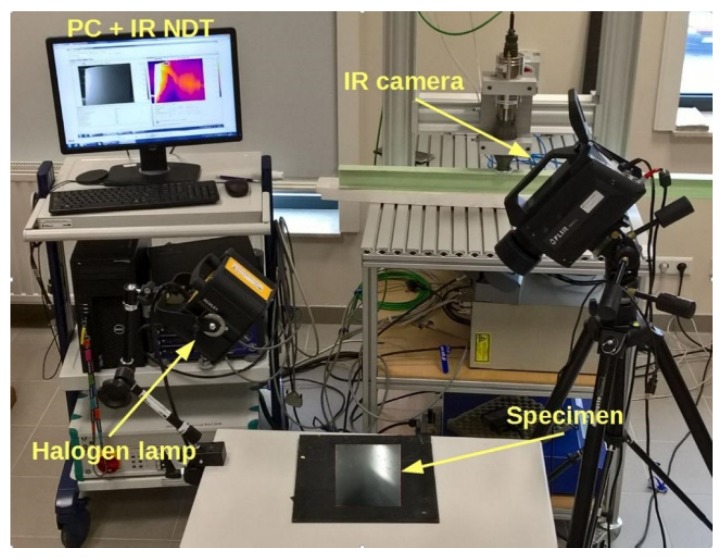
Non-contact active-IRT analysis for debond detection in the ACCS.

In the study, the non-contact transient IRT test is considered for the NDE of hidden debonds in the target structure. In IRT, a surface of the analysed ACCS specimen is stimulated by a heat pulse and the thermal response of the material is measured and analysed. The response in the form of thermal decay after the heat-flux contains information about sub-surface material defects (if any). After the heat pulse, the temperature decrease rate is different for damaged and undamaged areas. The idea of transient IRT measurements uses a halogen lamp for generating the thermal source, an IR camera to capture the thermal images and a computing device for the recorded thermal image data processing. The experimental set-up in the laboratory is described in Figure 4. In the experiment, the thermal source from the halogen lamp is directly applied to the ACCS surface and the thermal response of the structure is captured by a FLIR^(R)^: SC-6540 IR camera equipped with a cooled indium antimonide detector. The recorded sequences of thermal images (i.e., thermograms) are processed with an IR-NDT software from the Automation Technology.

## 3. Finite Element Analysis

Numerical simulations of guided wave propagation in the sample ACCS (25 cm × 25 cm × 0.7 cm) panel have been carried out using the explicit and implicit finite element analysis codes in ABAQUS. In the process, the ACCS panel is modelled in the explicit-code and the PZTs are modelled using the implicit-code. The standard explicit co-simulation technique is assigned to link the explicit and implicit analysis codes [9,12].

In ABAQUS, the eight-noded C3D8R standard explicit linear brick-elements are used for the modelling of ACCS. The element size for the CFRC and epoxy-adhesive layers are assigned as (0.05 cm × 0.05 cm × 0.025 cm) and (0.05 cm × 0.05 cm × 0.01 cm), respectively. The homogenised elastic properties of the CFRC laminate and the adhesive layer are calculated as per the Vinson and Sierakowski (1993) in [29]. The temperature-dependent material properties are then obtained as per Chamis (1984) in [30]. The engineering properties of the layered ACCS for the temperature-dependent numerical simulations are presented in Table 2. 

**Figure 5:**
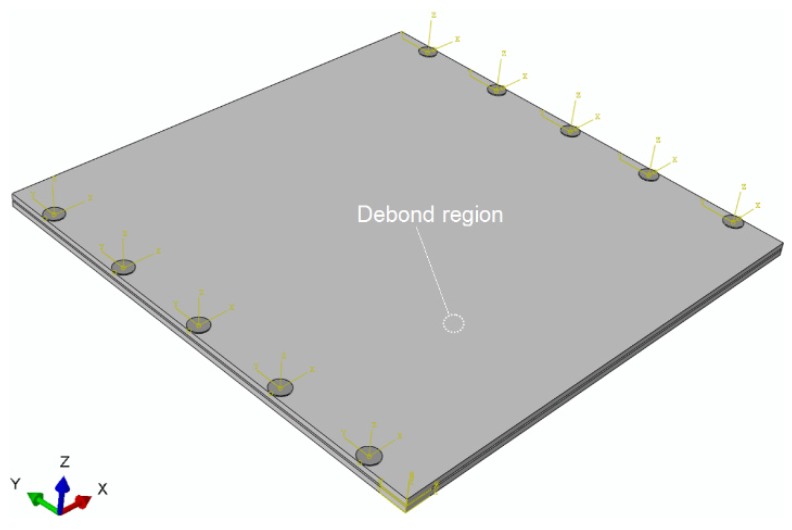
A numerical model of the ACCS sample for the simulation of guided wave propagation.

In PZT models (1 cm dia. and 0.04 cm thin), the eight-noded C3D8E standard implicit linear piezoelectric brick elements of size (0.05 cm × 0.05 cm × 0.025 cm) are assigned. The C3D8E elements offer the electro-mechanical-coupling property of PZTs. In the actuator PZTs, the driving signals (voltage) are applied to the top-surface nodes and 0-voltage is assigned to the bottom nodes. Whereas for the sensor-PZTs, the propagated signals (voltage) are registered at the top nodes and 0-voltage is applied to the bottom nodes. The fixed boundary conditions are assigned to the ACCS edges and to the PZT top-surfaces that implies no rotations and translations along the X,Y and Z directions. In the simulation, the NCE51 PZT (actuators/sensors) properties are considered as per the manufacturer’s (Noliac^(R)^) information:

mass-density, *ρ* = 7210 [kg/m^3^],

elastic-stiffness, $\left[C\right]=\left[\begin{array}{cccccc} 132 & 8.8 & 90.5 & 0 & 0 & 0\\ 0 & 132 & 90.5 & 0 & 0 & 0\\ 0 & 0 & 121 & 0 & 0 & 0\\ 0 & 0 & 0 & 20.2 & 0 & 0\\ 0 & 0 & 0 & 0 & 20.2 & 0\\ 0 & 0 & 0 & 0 & 0 & 22.6 \end{array}\right][\mathrm{GPa}],$,

charge-constant, $\left[e\right]=\left[\begin{array}{cccccc} 0 & 0 & 0 & 0 & 13.35 & 0\\ 0 & 0 & 0 & 13.35 & 0 & 0\\ -6.16 & -6.16 & 15.77 & 0 & 0 & 0 \end{array}\right][C/m^{2}]$ and

permittivity, $\left[\varepsilon \right]=\left[\begin{array}{ccc} 1947 & 0 & 0\\ 0 & 1947 & 0\\ 0 & 0 & 1911 \end{array}\right]\times 8.85\times 10^{-12}[F/m],$,

The numerical model of the sample ACCS with the selected network of PZTs (actuators/sensors) is described in Figure 5. The simulation is further extended for the variable debond sizes (φ = 0.4 cm, 0.8 cm (reference), 1.2 cm, 1.6 cm, 2.0 cm, and 2.4 cm) and for a series of ambient temperature conditions (−60 °C, −40 °C, 0 °C, 20 °C (reference), 40 °C, 60 °C, 80 °C and 100 °C). In all the simulation cases, the time-step of calculation is controlled to be 1e^−7^ < (minimum distance of any two connecting nodes/maximum accountable propagation velocity of the wave modes).

## 4. Results and Discussion

**Figure 6:**
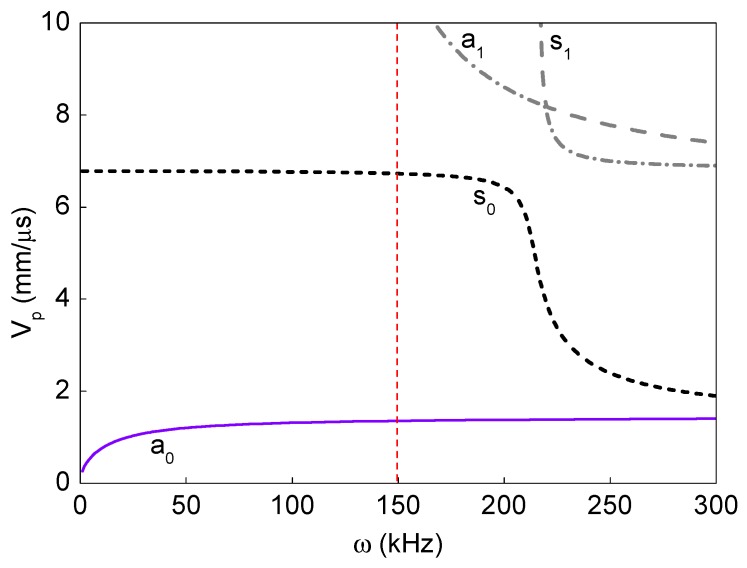
Dispersion curves for the guided wave propagation in the pristine ACCS at 20 °C.

Guided wave dispersion curves up to an operating frequency range are theoretically obtained based on the semi-analytical model described in [18] and the same formulations are not repeated here for brevity. The theoretically obtained phase-velocity dispersion curves for the ACCS presented in Figure 6 show the presence of two independent guided wave modes (primary anti-symmetric (a_0_) and primary symmetric ($s_{0}$) modes) at 150 kHz.

### 4.1. Numerical and Experimental Analysis of Debond Effects

**Figure 7:**
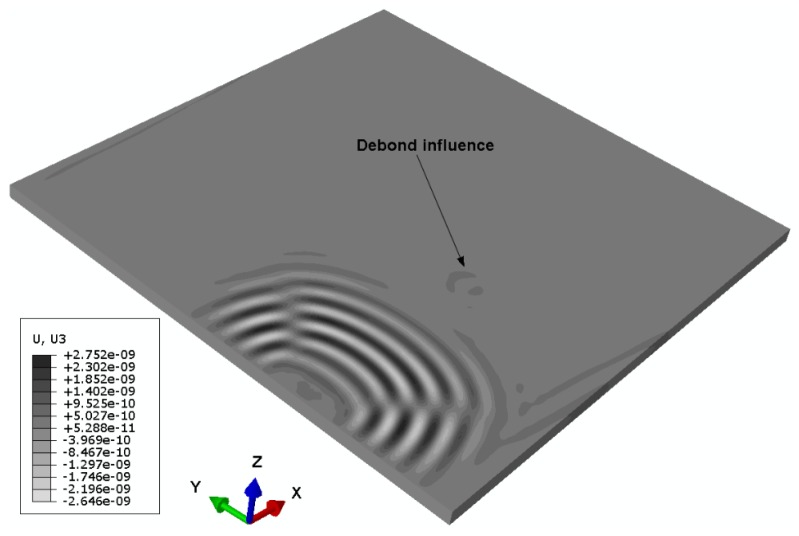
Waveform plot showing the debond influence on the guided wave propagation.

**Figure 8:**
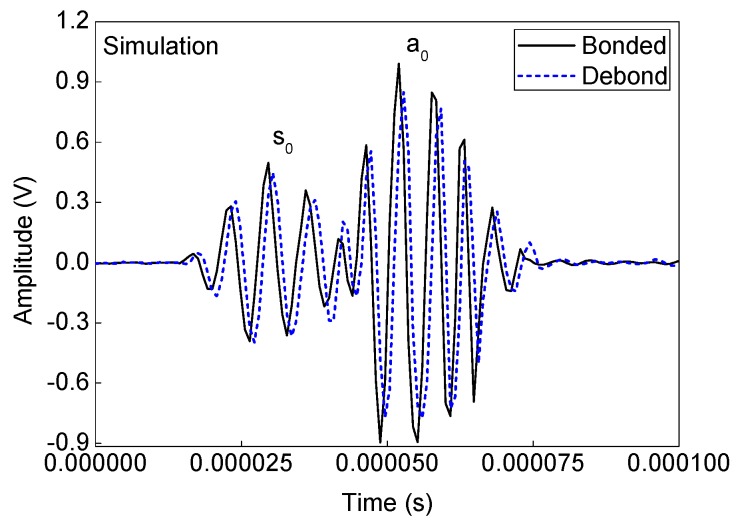
A typical comparison of bonded and debond influenced simulation signals.

Numerical simulation signals are obtained for the model described in Figure 5. A waveform plot from the simulation is shown in Figure 7, which clearly shows the debond influence on the guided wave propagation in the ACCS. In all the simulation signals, the wave modes are effectively identified and a comparison of bonded (pair: 7-8) and debond influenced (pair: 3-4) signals (Figure 2) at the reference temperature is presented in Figure 8. 

**Figure 9:**
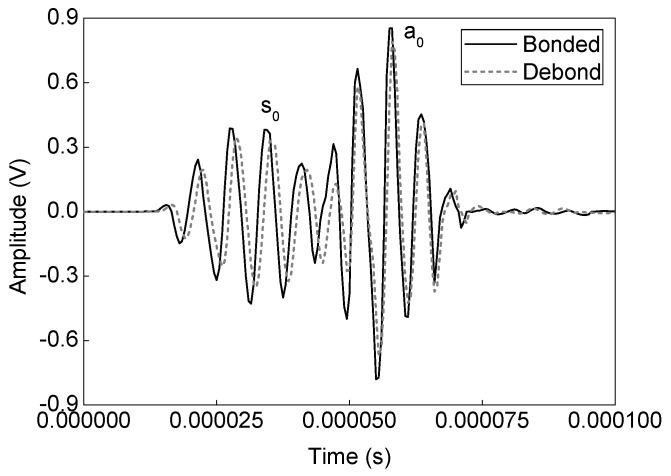
A typical comparison of bonded and debond influenced experimental analysis signals.

Experimental analysis signals are then collected from the assigned network of PZTs (Figure 2) on the sample surface. A comparison between the bonded (actuator–sensor pair: 7–8) and debond influenced (actuator–sensor pair: 3–4) signals at the reference temperature (i.e. 20 °C) are shown in Figure 9. In the signals, the ‘a_0_’ and ‘s_0_’ wave modes are accurately identified based on the dispersion curves in Figure 6. The results show that the presence of debond region in the ACCS significantly reduces the modal amplitudes as in the case of numerical simulation. Such reductions might have happened due to the reflections and major attenuation in the debond region. 

### 4.2. SHM Strategy for Debond Detection

In order to identify debonds in the ACCS, a debond index (DI) algorithm-based SHM strategy is proposed that uses the continuous wavelet transform (WT) of the guided wave signals from the assigned PZT sensors on the target structure. The algorithm calculates debond index values based on the difference in WT coefficient (WTC) magnitudes of the input signals at each pre-defined actuator– sensor combinations. The algorithm is prepared in the MATLAB environment that calculates a (10 × 10) matrix with 100 individual guided wave signals as inputs corresponding to the following combinations of actuator–sensor paths: path# 1–1, …, 1–10; path# 2–1, …, 2–10; 3–1; path# 3–1, …, 3–10; path# 4–1, …, 4–10; path# 5–1, …, 5–10; path# 6–1, …, 6–10; path# 7–1, …, 7–10; path# 8–1, …, 8–10; path# 9–1, …, 9–10; path# 10–1, …, 10–10 to detect debonds in the target structure (Figure 2). The debond index at any point $n_{i}(x,y)$ in the selected grid on the structure is defined as

(1) $$D_{i}(x,y)=\sum_{A=1}^{10}\sum_{S=1}^{10}\int_{t_{1}}^{t_{2}}{\left(WTC\right)}^{2}dt$$

where ‘*i* = 1, …, 100’ is the number of sensors configurations, ‘*D’* is the debond index, ‘*A*’ is the actuator number, ‘*S*’ is the sensor number and ‘*t_1_*’ is the time required for the ‘*a_0_*’ wave mode to travel from any actuator to a particular sensor, which is calculated from $t_{1}=d/V_{gA0}$ where ‘*V_g_a*_0_’ is the *a*_0_ mode velocity, ‘*d*’ is the actuator–sensor distance and $t_{2}=(t_{1}+t_{B})$, where ‘$t_{B}$’ is a width of time window considering the *a*_0_ mode in the signals. In the study, only the ‘*a*_0_’ modes in the guided wave signals are considered for debond detection owing to their lesser propagation speeds and high sensitivity against minor defects.

#### 4.2.1. Detection of Debonds Using Experimental Signals

**Figure 10:**
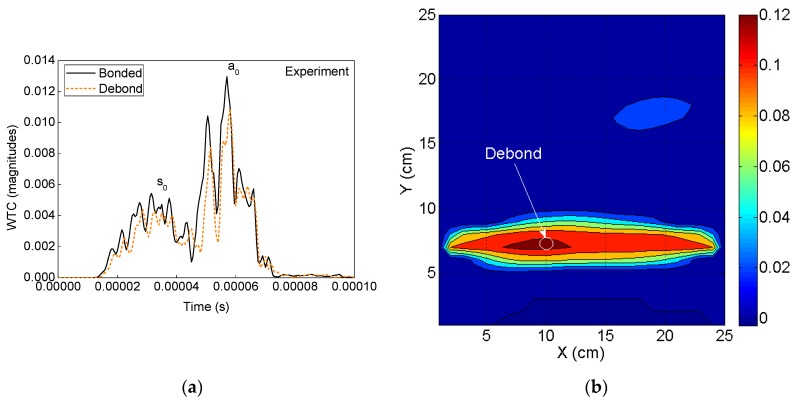
Experimental analysis: (**a**) A typical comparison of time domain WTs of the bonded and debond-influenced signals (Figure 9) and (**b**) debond index map showing the predicted debond location.

The debond region in the sample ACCS is identified using the experimental sensor signals to the proposed SHM strategy. In the process, the guided wave signals for the 10 actuator–sensor combinations are collected from the PZT network (Figure 2). The time domains WT of all the registered signals are then applied to the DI algorithm to calculate the differential WTCs, as shown in Figure 10a. The DI maps are obtained for the sample ACCS with a 0.8 cm φ debond region from the SHM framework. The map (contour pattern) is presented in Figure 10b that clearly indicates the debond location in the structure.

#### 4.2.2. Detection of Debonds Using Simulation Signals

**Figure 11:**
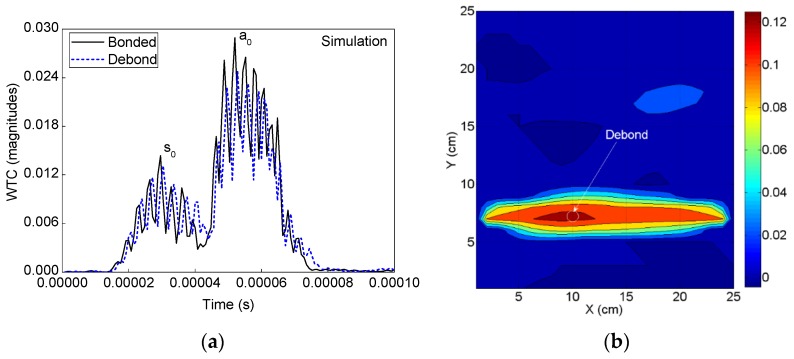
Numerical simulation: (**a**) A typical comparison between the WTs of bonded and debond-influenced signals (Figure 7) and (**b**) debond index map showing the identified debond location in the ACCS.

The debond region in the ACCS model is identified by using the simulated sensor signals to the SHM strategy. The WT of all the recorded signals from the PZT network are applied to the algorithm that calculates the differential WTCs as shown in Figure 11a. The DI map in Figure 11b is obtained from the SHM framework for the ACCS model with the 0.8 cm φ debond region. Good agreement is noticed between the experimentally and numerically obtained DI maps for the target ACCS.

### 4.3. Active IRT Analysis Based Non-Destructive Inspection of Debonds in the ACCS

The non-contact active IRT test results are obtained from the experimental analysis described in Figure 3, in order to verify the reliability of results from the proposed SHM strategy (Figure 10b and Figure 11b). In the IRT measurement, the thermograms are recorded for a duration of five seconds after thermal excitation. Further, in each measurement point, the polynomials approximating recorded signals are calculated. This method, called thermographic signal reconstruction [31], is one of the most widely used techniques. The evolution of the measured temperature is fitted to an n degree polynomial as

(2) $$T\left(t\right)=a_{0}+a_{1}t^{1}+\ldots +a_{n-1}t^{n-1}+a_{n}t^{n}$$

**Figure 12:**
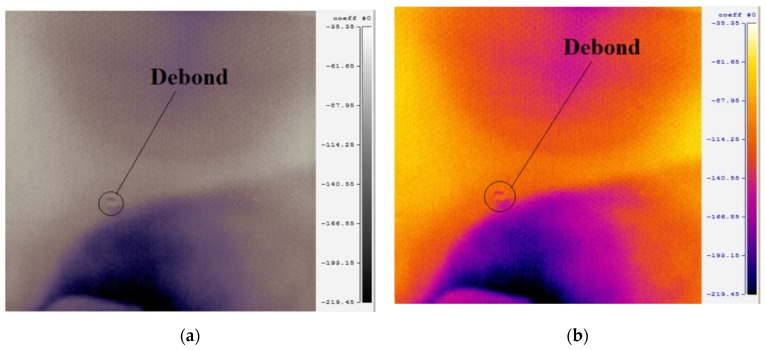
Debond localization maps corresponding to the selected polynomial coefficients for the recorded thermograms in (**a**) grey-scale and (**b**) colour-scale showing the actual debond location in the sample ACCS.

Analyzing polynomial coefficients and/or derivatives of the polynomials it is possible to observe the thermal propagation difference between pixels corresponding to damaged and undamaged areas. The maps of the selected polynomial coefficients are presented in Figure 12 for two different colour patterns. The maps distinctly represent the hidden debond location in the sample and fairly justifies the debond prediction potential of the proposed guided wave propagation based SHM framework that offers the possibility of in-service monitoring of such complex structures in safety-critical engineering applications.

### 4.4. Analysis of Debond Size and Temperature Variation Influence on the Difference in WTCs

**Figure 13:**
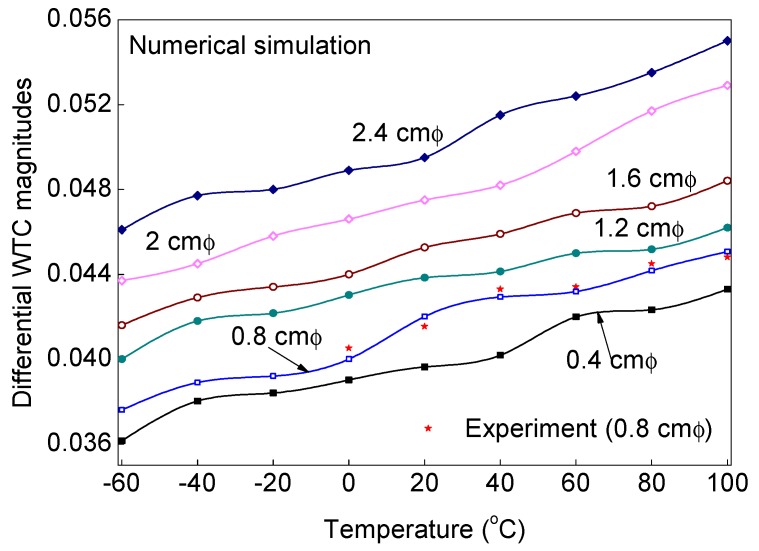
Change in WT-coefficient magnitudes between the bonded and debond (with different sizes) influenced signals under variable temperature conditions.

An extensive temperature-dependent study is experimentally and numerically carried out on the target ACCS to understand the influence of variable debond size and operating temperature on the differential WTC magnitudes (a critical component for the SHM strategy) of the propagated guided wave signals. In the study, the WTCs of experimental signals are obtained only for the signals collected from the ACCS sample with a 0.8 cm φ debond for a pre-defined range of operating temperature (0 °C, 20 °C, 60 °C, 100 °C) conditions. Whereas, the differential WTCs of simulation signals are separately obtained for ACCS models with 0.4 cm, 0.8 cm, 1.2 cm, 1.6 cm, 2.0 cm and 2.4 cm φ debond under a wide range of temperature conditions (−60 °C, −40 °C, 0 °C, 20 °C (reference), 40 °C, 60 °C, 80 °C and 100 °C), as per the practical scenarios. In all the cases, the sensor configurations in the PZT-network are maintained as per the details are given in Figure 2 and Table 1. The operating temperature versus differential WTC magnitude plots corresponding to different debond sizes is presented in Figure 13 for the typical case (WTC difference between the signals from path# 7–8 and path# 3–4). The results show the increase in the debond size and the ambient temperature significantly increases the differential *WTC* magnitudes of the guided wave signals. Good agreements between the experiment and simulation results are observed in all the study cases. 

### 4.5. Analysis of Temperature Variation Influence on the Debond Index Magnitudes

**Figure 14:**
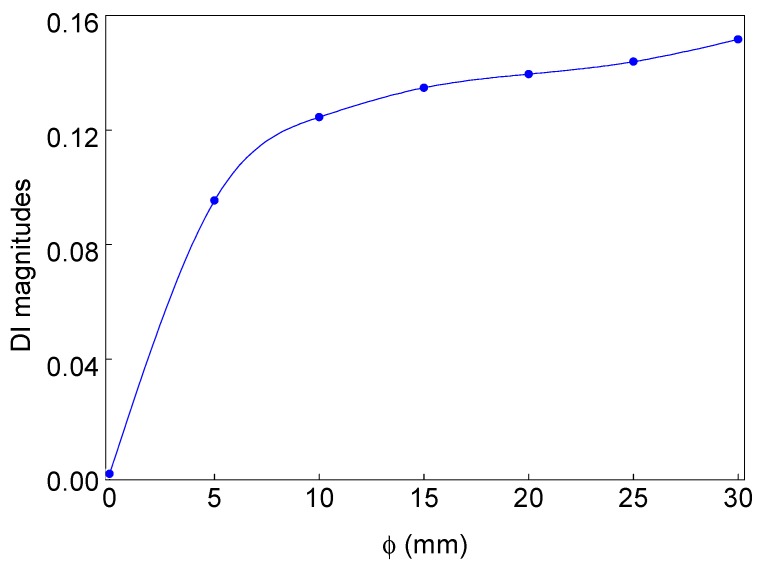
Change in debond index with the variation in debond size (φ).

The bond monitoring study is further extended for the analysis of debond size influences on the debond index magnitudes obtained from the proposed SHM strategy. In the process, the numerical sensor signals (Figure 2) are collected from the ACCS models with 0.4 cm, 0.8 cm, 1.2 cm, 1.6 cm, 2.0 cm, and 2.4 cm φ debond, respectively. WT of the collected signals are then applied to the monitoring framework and the corresponding debond index magnitudes are recorded. A debond size versus debond index magnitude plot is obtained and presented in Figure 14, which clearly shows the increase in debond size significantly increases the debond index magnitudes of the localization maps. Therefore, this strategy can be adopted for debond size prediction/characterization in a different type of composite structures under specified conditions (operating temperature, frequency, moisture-content, sensor configuration, type of PZTs).

### 4.6. Analysis of Temperature Variation Influence on the Debond Index Magnitudes

**Figure 15:**
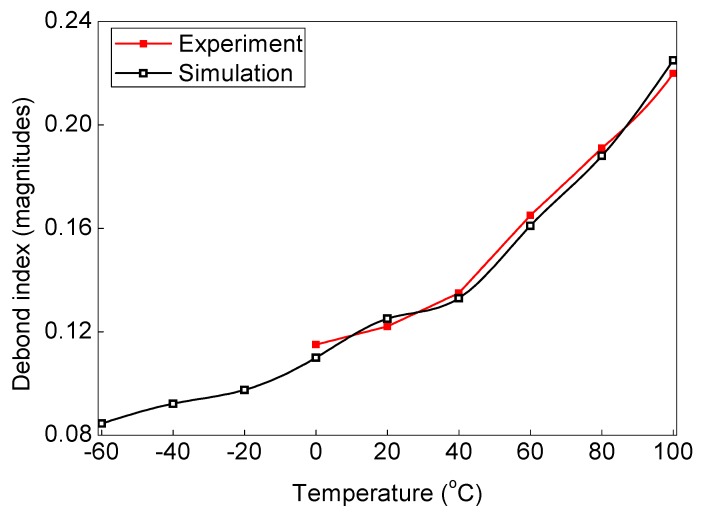
Change in debond index with the variation in ambient temperature.

In order to study the variable operating temperature influences on the debond index magnitudes, WT of the experimental (Figure 1) as well as simulation (Figure 5) signals are applied to the proposed SHM strategy. The experimental signals are collected for the ACCS sample with 0.8 cm φ debond under operating temperature: 0 °C, 20 °C, 60 °C, 100 °C. Whereas, the simulation signals are separately collected for ACCS models with 0.8 cm φ debond under operating temperature: −60 °C, −40 °C, 0 °C, 20 °C (reference), 40 °C, 60 °C, 80 °C, and 100 °C. The operating temperature versus debond index magnitude plots are obtained for the simulation and experimental data and presented in Figure 15. The results show the increase in ambient temperature significantly increases the debond index magnitudes in the localization maps. This increment is possibly related to a reduction in E-values with increasing temperature which can lead to ad hoc variations in wave mode amplitudes. A good agreement between the experiment and simulation results is observed. Therefore, this study can be adopted for the development of industry-grade SHM tools/frameworks for effectively identifying debonds under variable operating temperature conditions. 

Non-destructive analysis of adhesive bonds and a proposal of guided wave propagation-based reliable SHM framework for the ACCS is the prime focus of this paper. However, a more detailed investigation is required for a wide range of ACCSs and operating frequencies to develop robust industry-grade online monitoring strategies.

## 5. Conclusions

The work presented in this paper demonstrates the novel idea of using differential signal features of guided waves to localize and quantify the size of debond in adhesive-bonded composite structures. The constructed damage index has been shown to be a useful metric for capturing the variation in size of debond and is sensitive to the change in ambient temperature. Some of the important conclusions from the study can be summarized as follows

- The existence of debond at the adhesive layer in the ACCS reduces the amplitudes of propagating wave modes.
- Increase in the debond size and the ambient temperature significantly increases the a_0_ mode amplitudes and leads to an increase in amplitude difference between the bonded and debond influenced signals.
- The proposed SHM strategy is verified with the non-contact active IRT measurements and it has proven the debond detection potential in the ACCS.
- The proposed SHM strategy has further shown that the increase in debond size increases the debond index magnitudes of the localization maps, and the increase in ambient temperature also increases the debond index magnitudes in the localization maps.

However, a more detailed investigation on adhesive bonds in a different type of ACCS would be the future research scope to develop robust industry-grade online monitoring tools/strategies, which is ongoing research by the authors.

## Figures and Tables

**Table 1:** Coordinates (x,y) of the PZTs on the ACCS surface.

PZT (No.)	*X*-Coordinate (cm)	*Y*-Coordinate (cm)
1	0.5	2.5
2	24.5	2.5
3	0.5	7.5
4	24.5	7.5
5	0.5	12.5
6	24.5	12.5
7	0.5	17.5
8	24.5	17.5
9	0.5	22.5
10	24.5	22.5

**Table 2:** Temperature-dependent material properties of the ACCS.

Material	E_11_ (GPa)	E_22_ (GPa)	E_33_ (GPa)	G_12_ (GPa)	G_23_ (GPa)	G_13_ (GPa)	ν_12_	ν_13_	ν_23_	ρ (kg/m^3^)	Tmp (°C)
CFRC	73.95	73.95	11.17	4.26	4.02	4.02	0.04	0.37	0.37	1568	–60
	73.78	73.78	11.12	4.11	3.86	3.86	0.04	0.37	0.37	1568	–40
	73.53	73.53	10.7	3.95	3.71	3.71	0.04	0.37	0.37	1568	–20
	73.28	73.28	10.26	3.78	3.55	3.55	0.03	0.37	0.37	1568	0
	73.02	73.02	9.8	3.61	3.38	3.38	0.03	0.37	0.37	1568	20
	72.74	72.74	9.31	3.42	3.21	3.21	0.03	0.37	0.37	1568	40
	72.44	72.44	8.79	3.23	3.02	3.02	0.03	0.37	0.37	1568	60
	72.12	72.12	8.22	3.017	2.83	2.83	0.03	0.37	0.37	1568	80
	71.77	71.77	7.62	2.79	2.61	2.61	0.03	0.37	0.37	1568	100
Adhesive	4.82	4.82	4.82	1.71	1.71	1.71	0.4	0.4	0.4	1250	–60
	4.63	4.63	4.63	1.65	1.65	1.65	0.4	0.4	0.4	1250	–40
	4.45	4.45	4.45	1.59	1.59	1.59	0.4	0.4	0.4	1250	–20
	4.26	4.26	4.26	1.52	1.52	1.52	0.4	0.4	0.4	1250	0
	4.052	4.052	4.052	1.45	1.45	1.45	0.4	0.4	0.4	1250	20
	3.84	3.84	3.84	1.37	1.37	1.37	0.4	0.4	0.4	1250	40
	3.61	3.61	3.61	1.29	1.29	1.29	0.4	0.4	0.4	1250	60
	3.37	3.37	3.37	1.2	1.2	1.2	0.4	0.4	0.4	1250	80
	3.11	3.11	3.11	1.11	1.11	1.11	0.4	0.4	0.4	1250	100
